# Dysphonia as a sign of HPV laryngeal infection: a case report

**DOI:** 10.1186/1756-0500-7-898

**Published:** 2014-12-11

**Authors:** Carlos Eduardo Gama Longubuco, Helena Lucia Barroso dos Reis, Fernanda Sampaio Cavalcante, Carla Renata Petillo de Pinho, Nathalia Silva Oliveira, Alcina Frederica Nicol, Renata Quintella Zamolyi, Dennis de Carvalho Ferreira

**Affiliations:** Biologic Science Undergraduate Program - Microbiology Program, Salgado de Oliveira University, Rio de Janeiro, Brazil; Ginecology and Obstetrics Outpatient Care, Federal University of Espírito Santo (UFES), Vitória, Brazil; Paulo de Goes Microbiology Institute - Medical Microbiology Department, Federal University of Rio de Janeiro, Rio de Janeiro, Brazil; Oral Medicine Area, School of Dentistry, Veiga de Almeida University, Rio de Janeiro, Brazil; Laboratory of Interdisciplinary Medical Research (LIPMED), Oswaldo Cruz Foundation -Fiocruz, Rio de Janeiro, Brazil; Pathology Anatomy Service, Bonsucesso Federal Hospital, Rio de Janeiro, Brazil; CAPES Fellow (Bolsista da CAPES), Proc. n BEX 9203, CAPES Foundation, Ministry of Education of Brazil, Brasilia, Brazil; Abeu University Center, Belford Roxo, Rio de Janeiro, Brazil

**Keywords:** Dysphonia, Human papillomavirus, Vocal fold

## Abstract

**Background:**

Voice dysfunction or dysphonia may be associated with several clinical conditions. Among these, laryngeal human papillomavirus (HPV)-induced lesions should be considered as a possible causative factor. We report a case of dysphonia in a patient presenting with an HPV laryngeal lesion. We also discuss the clinical features of the disease, its histopathological findings, and treatment and rigorous follow-up.

**Case presentation:**

We report a case of laryngeal papilloma in a 29-year-old, Afro-descendant, male patient with dysphonia. He was a non-smoker and was not a drug user. Videolaryngostroboscopy revealed signs suggestive of pharyngolaryngeal reflux. The right vocal fold presented with a papillomatous aspect in the posterior third, which underwent excision. Histopathological examination showed a nodular lesion of the right vocal fold, conclusive of squamous papilloma with absence of malignancy.

**Conclusion:**

Patients presenting with persistent voice dysfunction or dysphonia should be investigated for possible laryngeal HPV infection. Diagnostic confirmation by HPV genotyping is important for follow-up of potential recurrence.

## Background

Voice dysfunction or dysphonia has widespread effects and has been frequently studied. Functional and neoplastic dysphonia is among the many causative factors; however, it may also be associated with neurological and systemic diseases, as well as other diseases such as oropharyngeal papilloma, which is a benign tumor caused by human papillomavirus (HPV). More than 200 HPV types have been described and 40 are capable of infecting the anogenital and oral epithelia. HPV may be clinically characterized by appearing in proliferative processes such as oral papilloma, commonly associated with low-risk genotypes 6 and 11. It has also been considered as the major etiological agent in development of neoplasia of the anogenital tract, such as cervical, anal, and penile cancer, as well as oral tumors, where there is a higher prevalence of high-risk genotypes 16 and 18 [1].

The World Health Organization (WHO) considers HPV infection to be an epidemic of high relevance for public health [2].

The most frequently observed oral manifestations associated with HPV infection there include: condyloma acuminatum, focal epithelial hyperplasia, verruca vulgaris, and oral squamous papilloma. These lesions are all benign and are normally associated with infection with low-risk HPVs, among which subtypes 6 and 11 are more prevalent [3, 4].

HPV infection may also be associated with oropharyngeal carcinoma development, among which, squamous cell carcinoma is the eighth most common type of cancer in men. Additionally, persistent virus infection, together with the use of alcohol and tobacco are predisposing factors for development of oropharyngeal cancer [3, 5].

One of the main complaints with these types of lesions is speech alteration (dysphonia), as reported in the present case, and deglutition (dysphagia) [6]. Ximenes Filho et al. have reported laryngeal papillomatosis, and 54.54% of patients presented with dysphonia, and 4.54% with dysphagia and bleeding [6].

Here, we describe a case of dysphonia in a patient diagnosed with vocal chord papilloma. This article addresses an uncommon issue of dysphonia related to HPV-induced lesions in an adult patient, highlighting the need for further investigation of dysphonia.

## Case presentation

A 29-year-old Afro-descendant, single male, undergraduate biological sciences student, who lives in Itaboraí-Rio de Janeiro (RJ) and works as a waiter, was referred by the stomatologist to the Department of Otorhinolaryngology of a private hospital, reporting dysphonia and hoarseness for 2 months.

He is homosexual and reported nonexclusive sexual partnerships in the previous year, and casual condom use during oral and anal intercourse. Regarding HPV infection, his partner already had virus-induced lesions in the anal region, although he had been treated. The patient denied smoking, social drinking, and illicit drug use. He had a verruca vulgaris in his right hand that disappeared without surgery or medication when he was a child. He reported being born by cesarean section.

Lesions were not observed during the intra and extra-oral physical examination, and neither was cervical lymphadenopathy noted. Oral hygiene was satisfactory and there was no biofilm retention, caries, or gingivitis. Body examination revealed a wart in the foot.

**Figure 1:**
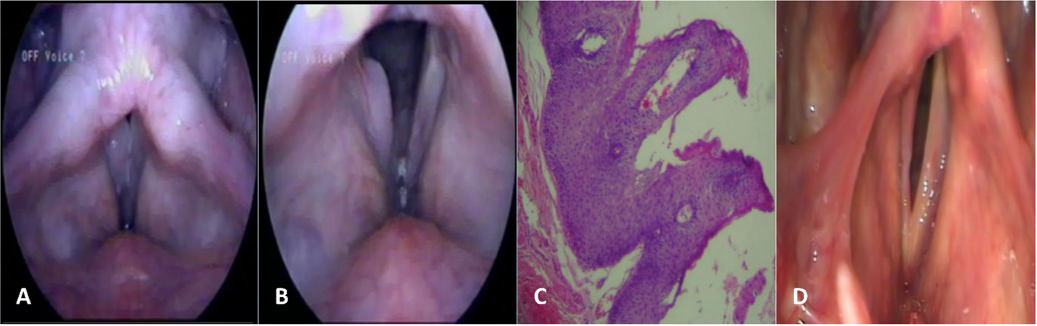
**Laringeal videolaryngostroboscopy along with Histopathology. (A)** Closed vocal fold. Normal anatomical pharyngolaryngeal sites **(B)** Open vocal fold. Suggestive signs of pharyngolaryngeal reflux and retrocricoid and interarytenoidal edema and presence of papillomatous lesion. **(C)** Histopathology image. **(D)** Videolaryngostroboscopy after excision.

Videolaryngostroboscopy presented signs suggestive of pharyngolaryngeal reflux, and retrocricoid and interarytenoidal edema. Remaining unchanged anatomical pharyngolaryngeal sites were noteworthy (Figure 1A,B) There was formation of a papillomatous aspect in the posterior third and part of the middle third of the right vocal fold. It was also observed normal mobility, reduced mucosal wave activity, normal frequency, and low-amplitude. There was a mild lateral supraglottic constriction, and absence of posterior rhinorrhea. Examinations for sexually transmitted diseases (STDs), including HIV, syphilis, hepatitis B and C, and gonorrhea were performed with negative results.

After routine preoperative examinations, the patient was referred for surgical excision of the nodular lesion of the right vocal fold. Histopathological examination showed a nodular lesion of the right vocal fold, with several small, firm and elastic brown tissue fragments, with some papillomatous areas measuring 1.7 × 0.8 × 0.2 cm, conclusive of squamous papilloma with absence of malignancy (Figure 1C). Polymerase chain reaction (PCR) detected HPV type 6.

After surgical excision of the squamous papilloma on the right vocal fold, the patient was prescribed analgesic and anti-inflammatory agents. Among the prescribed restrictions there was absolute voice rest and ingestion of a liquid and pasty diet, preferably cold or frozen. One week after the procedure, the patient still had difficulty speaking owing to postoperative inflammation. He was prescribed continuous vocal rest and anti-inflammatory agents. After a period of 3 months, another videolaryngostroboscopy was performed, which revealed normal appearance of the epiglottis in the supraglottic region and good mobility, and unchanged aryepiglottic and ventricular folds. In the glottis we observed the right vocal fold with posterior retraction in the middle third, hyperemia in the vocal fold, glottic closure with formation of fusiform average posterior glottis, preserved vocal fold mobility and vertical vocal fold level. It was also observed a non-symmetrical vocal fold vibration, reduced wave mucosa, absent adynamic segments; normal supraglottic activity; free pyriform sinus; and arytenoids with normal appearance.

Videolaryngostroboscopy concluded that, after excision in the glottis, there was a decline in the middle third of the right vocal fold, formation of spindle glottis phonation, right vocal fold hyperemia, and reduction of the mucosal wave in the right vocal fold (Figure 1D).

Upon the diagnostic appearance presented by postoperative videolaryngostroboscopy, the patient was referred to a phonoaudiologist to perform vocal rehabilitation. He is currently undergoing clinical follow-up.

## Conclusions

Dysphonia may be indicative of vocal cord papilloma; therefore, its presence deserves a thorough investigation to establish a proper diagnosis. A study by Ximenes Filho et al. of laryngeal papillomatosis found that 54.54% of patients had dysphonia and 4.54% had dysphagia and bleeding [6]. This was corroborated in the present case that presented with a complaint of dysphonia.

Populations with risky behavior, such as unprotected sexual intercourse, and nonexclusive partners, are more exposed to HPV infections. Our patient was in the men who have sex with men (MSM) at-risk population. He admitted to having a sexual partner who had already presented with condylomatous anal injury. This may have contributed to the emergence of HPV laryngeal infection, because our patient has maintained relationships with the previously infected individual, and the practice of fellatio is positively associated with HPV infection [3]. A literature review has shown that 57% of MSM had HPV DNA; 26% infected with low-risk HPV and 26% with high-risk HPV [7]. Additionally, the HPV infection in this population also increases the risk of HIV infection and other STDs, as well as the appearance of oral and laryngeal lesions [8].

Laryngeal papillomatosis is a disease that is characterized by a verruca, whose main etiological agent is HPV. The injury is typically diagnosed as benign squamous papilloma and can lead to discomfort in speaking, affecting mainly the vocal folds, epiglottis and vestibular folds, with possible recurrence [9–11].

Histopathological diagnosis indicated a nodular lesion on the vocal folds, characterizing papillomatosis that was confirmed as squamous papilloma with absence of malignancy. This corroborates the majority of studies in which the main viral types found in this injury are HPV 6 and 11, which are considered low-risk oncogenic types [3].

Histopathological diagnosis indicated squamous papilloma. In addition, PCR based molecular biology techniques by PCR confirmed HPV 6 infection in this particular case.

There is evidence to suggest the need to improve prevention strategies in order to reduce the prevalence of STDs and related complications [12].

In conclusion, patients presenting with persistent voice dysfunction or dysphonia should be investigated for possible laryngeal HPV infection. Diagnostic conformation by HPV genotyping is important for follow-up of potential recurrence.

## Consent

Written informed consent was obtained from the patient for publication of this case report and accompanying images. A copy of the written consent form is available for review by the Editor-in-Chief of this journal.
